# Impairment of T Cell Function in Parasitic Infections

**DOI:** 10.1371/journal.pntd.0002567

**Published:** 2014-02-13

**Authors:** Vasco Rodrigues, Anabela Cordeiro-da-Silva, Mireille Laforge, Ali Ouaissi, Khadija Akharid, Ricardo Silvestre, Jérôme Estaquier

**Affiliations:** 1 CNRS FRE 3235, Université Paris Descartes, Paris, France; 2 Parasite Disease Group, Instituto de Biologia Molecular e Celular, Universidade do Porto, Porto, Portugal; 3 Departamento de Ciências Biológicas, Faculdade de Farmácia da Universidade do Porto, Porto, Portugal; 4 Département de Biologie, Faculté des Sciences Aîn-Chock, Université Hassan II-Casablanca, Casablanca, Maroc; 5 Université Laval, Centre de Recherche en Infectiologie, Québec, Canada; Federal University of São Paulo, Brazil

## Abstract

In mammals subverted as hosts by protozoan parasites, the latter and/or the agonists they release are detected and processed by sensors displayed by many distinct immune cell lineages, in a tissue(s)-dependent context. Focusing on the T lymphocyte lineage, we review our present understanding on its transient or durable functional impairment over the course of the developmental program of the intracellular parasites *Leishmania* spp., *Plasmodium* spp., *Toxoplasma gondii*, and *Trypanosoma cruzi* in their mammalian hosts. Strategies employed by protozoa to down-regulate T lymphocyte function may act at the initial moment of naïve T cell priming, rendering T cells anergic or unresponsive throughout infection, or later, exhausting T cells due to antigen persistence. Furthermore, by exploiting host feedback mechanisms aimed at maintaining immune homeostasis, parasites can enhance T cell apoptosis. We will discuss how infections with prominent intracellular protozoan parasites lead to a general down-regulation of T cell function through T cell anergy and exhaustion, accompanied by apoptosis, and ultimately allowing pathogen persistence.

## Introduction

Infections caused by the intracellular protozoa *Leishmania* spp., *Trypanosoma cruzi*, *Plasmodium* spp., and *Toxoplasma gondii* are associated with high morbidity and a heavy economic toll. These unicellular eukaryotes display complex life cycles whose successful completion relies on shuttling between different hosts. Particular selective pressures during host–pathogen coevolution shaped the developmental program of each parasite, giving rise to distinct clinical conditions (Box 1).

**Box 1. Developmental Programs of Intracellular Parasitic Protozoa in the Mammalian Host and Associated Clinical Conditions**

The kinetoplastids *Leishmania* spp. and *T. cruzi* and the apicomplexans *Plasmodium* spp. all rely on insect vectors for transmission to the mammalian host. After deposition in the dermis through the bites of infected sand flies, *Leishmania* parasites reside inside host phagocytes and, depending on the infecting species, can either cause localized cutaneous lesions (e.g., *L. major*) or visceral leishmaniasis (*L. donovani*, *L. infantum*), a chronic disease characterized by amastigote accumulation in visceral compartments such as the spleen or the liver.

*T. cruzi* metacyclic trypomastigotes are transmitted by the reduviid bug and cause an acute infection that lasts some weeks and is characterized by systemic infection of multiple host nucleated cells, within which the parasite persists in a cytoplasmic location. Development of adaptive immunity restricts parasite numbers and signals the beginning of chronic infection, which may persist for the life of the host. About two-thirds of the infected patients will never be afflicted by clinical disease during the chronic phase, while the remaining may develop chagasic cardiomyopathy or digestive complications such as megacolon or megaesophagus, usually 10 to 30 years after the initial infection.

*Anopheline* mosquitoes transmit *Plasmodium* sporozoites to the dermis of the host, initiating a developmental program that starts with parasite migration to the liver. The liver stage of infection is clinically silent but results in remarkable replication of the merozoite form inside hepatocytes. Merozoite egress from hepatocytes and infection of erythrocytes initiates the blood stage of infection and is responsible for the pathological sequelae that are typically associated with malaria, which include acidosis, anaemia, and cerebral malaria.

The apicomplexan *T. gondii* can infect humans through ingestion of undercooked meat containing viable tissue cysts or water contaminated with parasite oocysts. An early acute phase, which usually passes unnoticed or causes mild flu-like symptoms, is characterized by remarkable parasite dissemination in the body due to the virtually unlimited host cell range of the tachyzoite form. Strong pressure posed by adaptive immunity induces parasite differentiation to semidormant bradyzoites that form tissue cysts in the brain and muscle, initiating chronic infection that may last for the life of the individual. Complications arise in the case of acquired immunodeficiency and manifest as toxoplasmic encephalitis.

Protective immunity against parasitic infection is critically dependent on the development of a multifunctional T cell response that directly kills infected cells or induces phagocyte activation to destroy intracellular parasites [1]–[3]. As blood or tissue pathogens, their transmissibility to the insect vector or definitive host is low, and thus these pathogens devised strategies to dampen the T cell response and increase the time available for parasite transmission [4].

After breaching epithelial barriers, intracellular protozoa rapidly deploy strategies to resist innate mechanisms employed by infection site–recruited immune cells, such as macrophages or dendritic cells (DCs) [5], [6]. These cells are also responsible for the transition between innate immunity and the onset of the adaptive response. As such, inhibiting the signals emanating from antigen-presenting–cells (APCs) represents an ingenious strategy to delay or hamper T cell responses [7], allowing rapid parasite replication and dissemination during the acute stage of infection. Nevertheless, adaptive immunity eventually develops and is generally associated with control of acute parasite infection [8]–[10]. Yet, even in the presence of a robust T cell response, complete pathogen eradication is rarely achieved, signalling the onset of chronic infection, which may remain clinically silent throughout the host's life or give rise to complications several years after primary infection. Chronic parasite persistence has a profound impact on the effector capacity of T cells, inducing their gradual loss of function in a phenomenon known as T cell exhaustion [11].

Spanning both acute and chronic stages of infection is the programmed death of T cells, a homeostatic mechanism that ensures the elimination of most specific T cells after clearance of a foreign threat, yet allows the survival of a small number of memory cells capable of long-term, antigen-independent survival [12]. However, by interfering with the apoptotic T cell process, parasites may subvert the mechanisms of memory formation and reduce the numbers of specific T cells available to fight the pathogen in the long term [13].

Here, we review the current understanding of how intracellular protozoan parasites subvert the host T cell immunity during the full length of their developmental program within the mammalian host, through mechanisms that favour the occurrence of T cell anergy, exhaustion, and apoptosis.

### Subverting the Signals Required for T Cell Activation Results in Delayed or Anergic T Cell Responses

T cell anergy was initially described as a state of non-responsiveness induced in vitro at the time of T cell stimulation, via T cell receptor (TCR), in the absence of a costimulatory signal [14]. Lack of costimulation results in defective nuclear recruitment of the transcription factors required to initiate *IL2* transcription [7]. In this sense, anergy is usually induced in T cells that bind their cognate peptide-MHC complexes displayed in the surface of not fully mature APCs, hence unable to provide adequate costimulation [15]. In alternative, T cell anergy may be induced after expression of immunomodulatory molecules by APCs, including the ATP-catabolizing enzymes CD39 and CD73 or the tryptophan-metabolizing enzyme indoleamine-2,3-dioxygenase (IDO) [16]. These observations link the requirement of an inflammatory and/or immunogenic environment to efficient T cell activation (Table 1).

**Table 1:** T cell anergy versus T cell exhaustion.

Unresponsive state	Differentiation state affected	Driving forces	General characteristics of the unresponsive state
T cell anergy	Naïve/Effector	Impaired antigen presentation	Impaired activation and proliferation
		Reduced costimulation	Defective differentiation and effector function
		Expression of immunomodulatory molecules (IDO, CD73, CD39)	Apoptosis
		Regulatory cytokines (IL-10, TGF-β)	
T cell exhaustion	Effector	Antigen persistence, chronic activation	Progressive impairment of effector function
		Regulatory cytokines (IL-10, TGF-β)	Expression of inhibitory receptors (PD-1, TIM-3, LAG-3)
		Suboptimal priming (while in the naïve state)	Decreased expression of common γ chain cytokine receptors
			Apoptosis

Even though the infection site and the anatomical location of acute replication differ among the pathogens discussed here (Box 1), intracellular parasites share similar mechanisms to disturb the functions of APCs and preclude effective T cell priming during the acute phase of infection. For instance, in mice and humans, acute toxoplasmosis is associated with a transient, anergic-like suppression of T cell function [17]–[19]. In vitro studies unveiled the ability of *T. gondii* to down-modulate antigen presentation and costimulatory molecules in the infected APC [20]–[22]. Furthermore, a strong association between reduced DC-derived signals and decreased magnitude of the specific CD8 T cell response was observed after infection of mice with tachyzoites of the lethal RH strain, which is capable of remarkable dissemination through host tissues within the first few days after infection [19].

In murine *L. donovani* infection, parasites colonize the liver and quickly replicate, but hepatic infection self-resolves within one month [10]. A recent study found that LIGHT (a TNF superfamily ligand) signalling through the lymphotoxin-beta receptor (LT-βR) was detrimental to the onset of the specific CD4 T cell response, which may explain the rapid increase in hepatic parasite numbers during the first week after inoculation. An antibody therapy blocking LT-βR engagement by LIGHT resulted in increased TNF production by hepatic CD4 T cells, faster granuloma maturation and decreased hepatic parasite loads [23]. In contrast with the liver, the spleen is the site of chronic parasite persistence during murine visceral leishmaniasis. After *L. donovani* infection in mice, the expansion and activation of splenic CD8 T cells is delayed and lower in magnitude, when compared with a prototypical acute infection [24]. A similar delay in the onset of the splenic CD8 response was found during murine *T. cruzi* infection [25].

Increased expression of immunomodulatory molecules in APCs has also been noted in parasite infections [26]–[28]. Dendritic cells expressing ATP-metabolizing enzymes or IDO emerge in the local lymph nodes shortly after intradermal infection of mice with *L. amazonensis* and *L. major*, respectively. These DCs were poor inducers of T cell proliferation and, importantly, the T cell response could be restored by blocking the adenosine receptor A_2B_ or IDO [26], [27]. Interestingly, loss of IDO activity was recently proposed as a biomarker for the restoration of the immune response in treated leishmaniasis patients [29].

The liver stage of *Plasmodium* infection is very silent, both clinically and immunologically, possibly due to a lack of recognition of the intrahepatocytic merozoite by innate immunity [30]. After deposition in the dermis, most sporozoites do not reach the blood stream but are instead conveyed to the local lymph nodes and digested inside DCs [31]. In mice inoculated with irradiated sporozoites, CD8 T cells, primed by DCs in the skin-draining lymph nodes, are able to migrate to the liver, recognize infected hepatocytes, and provide protection [32]. Similarly, mice given prophylactic chloroquine at the time of live sporozoite inoculation are protected against subsequent challenge [33]. The success of both immunization strategies seems to advent from the development of a robust intrahepatic CD8 T effector/memory response associated with high IFN-γ production [34]. Thus, efficient CD8 T cell priming can occur during *Plasmodium* infections, and it is important to gain further knowledge of the properties of the activated/matured APCs generated with these immunization strategies, with the aim of optimizing vaccine design.

Contrasting with hepatic infection, the blood stage of malaria is noticeably immunogenic and, conversely, immune evasion mechanisms mediated by *Plasmodium* become apparent [30]. Splenic DCs recognize and internalize *Plasmodium*-infected red blood cells (pRBCs) but fail to stimulate T cells [35], [36]. This ability correlates with strain lethality [37] and is possibly caused by a systemic DC activation that occurs very early after inoculation (1 to 4 days), before the peak in parasitemia (days 4 to 7). As a consequence, presentation of parasite antigens is short-lived, as activated DCs become unable to phagocytose pRBCs, compromising T cell activation [38].

Parasite-derived molecules may directly inhibit T cell activation. Trans-sialidase, a glycoside hydrolase shed by *T. cruzi*, sialylates the surface of activated CD8 T cells, reducing their affinity to cognate peptide-MHC and decreasing their cytotoxicity [39]. Inhibition of trans-sialidase restored the CD8 T cell function and increased mice survival [39]. In addition, AgC10, a *T. cruzi* GPI-anchored mucin, binds to L-selectin in the surface of T cells and, by interfering with the phosphorylation of TCR-associated signal transducers, is capable of blocking *IL2* transcription, rendering T cells anergic during acute infection in mice [40].

Although anergy is viewed as a process regulating the initial phase of the T cell response, the emergence of regulatory T cells (Tregs) expressing immunomodulatory molecules, such as CTLA-4, IDO, or ATP-metabolizing enzymes, during parasite infection may contribute to sustaining T cell anergy during the chronic phase [41]–[44].

### Chronic Parasite Infection Leads to Exhaustion of Specific T Cells

Immune exhaustion corresponds to a loss of effector function of antigen-experienced T cells that occurs in a progressive manner, starting with decreased proliferative ability, IL-2 production, and cytotoxic function, followed by an incapacity to produce IFN-γ and TNF-α, and culminating with physical deletion at terminal stages [11], [45]. Exhausted T cells present high and sustained expression of inhibitory molecules such as programmed death-1 (PD-1), T-cell immunoglobulin, and mucin domain-containing protein-3 (TIM-3) and lymphocyte-activated gene-3 (LAG-3) [11] (Table 1).

T cell exhaustion associated with chronic infection was initially reported in viral models as specific CD8 T cells that failed to produce cytokines [46]. Recent work conjectured a similar pattern for chronic parasitic infections [47].

The occurrence of T cell exhaustion in viral models has been classically associated with concomitant high and persistent antigen levels [11]. In contrast, chronic parasite infections are characterized by lower pathogen burden, which is generally tissue-restricted, suggesting alternative driving forces in the induction of T cell exhaustion. For instance, acute *T. gondii* infection is usually controlled by the development of adaptive immunity, leading to parasite encystation and latency (Box 1). Nevertheless, this does not preclude subsequent exhaustion of CD8 T cells. Indeed, infection of C57Bl/6 mice with *T. gondii* cysts of the ME49 strain causes death in 7 weeks, associated with parasite reactivation in the brain and concurrent with decreased numbers of brain-infiltrating CD8 T cells and their reduced production of IFN-γ and granzyme B, an indication of cellular exhaustion [48]. Increased PD-1 expression accompanied T cell exhaustion, and a treatment blocking the PD-1/PD-L1 pathway resulted in reinvigorated T cell function and prevented animal demise [48], [49]. Recent data suggests that the CD40/CD40L axis plays a crucial role in the rescue of exhausted CD8 T cells in the context of α-PD-L1 therapy [1]. Importantly, reinvigoration of the CD8 T cell response through CD40L-CD40 signalling occurred not only in a CD8-intrinsic manner, but also by boosting CD4 helper cell function through induction of increased production of IL-21 [1], a cytokine previously shown to alleviate CD8 T cell exhaustion in viral models [50], [51].

In murine *L. donovani* infection, splenic CD8 T cells exhibit exhaustion around 4–5 weeks after inoculation, with reduced production of IFN-γ, TNF, and granzyme B [24]. PD-1 expression in parasite-specific CD8 T cells and PD-L1 expression in splenic DCs paralleled the decrease in T cell function and blocking PD-1/PD-L1 interactions could reduce splenic parasite burden [24]. In cutaneous leishmaniasis caused by *L. mexicana*, expression of PD-1 in peripheral blood CD8 T cells correlates with lesion severity being found in patients with diffuse but not localized lesions [52].

In contrast, parasite-specific CD8 T cells do not undergo functional exhaustion after mice infection with *T. cruzi*. Furthermore, after drug cure, CD8 T cells adopted a central memory phenotype and protected against reinfection [53], a finding at odds with the view that exhausted T cells are dependent on antigen persistence and lost after antigen removal [11]. However, early studies in human patients affected by chronic Chagas disease evidenced a functional impairment of T cells that correlated with severity of cardiac pathology [54]–[56]. The reason for this dichotomy may lie in the much longer timeframe of human infection (1–2 years in mice versus decades in humans), presumably having a more severe impact on the functionality of T cells.

In line with findings in chronic viral infection [57]–[59], during parasitic disease, not only CD8 T cells are subjected to functional exhaustion. Parasite-specific splenic CD4 T cells up-regulate PD-1 and LAG-3 and become exhausted by day 30 after mice infection with *P. yoelii*–infected RBCs [60]. Simultaneous blockade of PD-1 and LAG-3 increased the numbers of multifunctional CD4 T cells that produce IFN-γ, TNF, and IL-2 and accelerated parasite clearance [60]. In contrast, blockade of either PD-1 or LAG-3 alone had only modest effects on the recovery of functional CD4 T cells or decrease in parasitemia, suggesting that inhibitory receptors may play independent roles in the induction and/or maintenance of the exhausted state and that combined therapies might be more efficient in improving T cell fitness [61].

Importantly, caution has to be taken when classifying exhausted CD4 T cells based solely on the expression of PD-1. A recently described CD4 T cell subset, termed T follicular helper cells (Tfh), which is essential for B cell–mediated immunity, is characterized by the expression of PD-1 in association with the B cell follicle-homing chemokine receptor, CXCR5. In this context, it is interesting to note that α-PD-L1/α-LAG-3 therapy dramatically increased *P. yoelli*–specific humoral responses and the numbers of germinal centre B cells, presumably due to the accompanying increase in the number of Tfh cells. These findings suggest that therapeutic blockade of inhibitory receptors during chronic parasite infection may have beneficial effects that extend beyond the recovery of exhausted T cells. Finally, increased expression of PD-1 and LAG-3 has been recapitulated in T cells from human patients infected with *P. falciparum* [60], [62].

It is worth mentioning, however, that despite their deleterious role in sustaining T cell exhaustion during chronic infections, inhibitory receptors protect host tissues during acute infection by dampening potentially pathogenic T cell responses. During acute *T. cruzi* infection in mice, ablating PD-1 signalling augments cardiac inflammation due to increased infiltration of activated CD4 and CD8 T cells [63]. Similarly, blocking the PD1/PD-L1 pathway during *P. berghei* ANKA infection promotes CD8 T cell infiltration in the brain and augments the incidence of experimental cerebral malaria (ECM) in otherwise ECM-resistant Balb/c mice [61]. Thus, signalling through inhibitory receptors appears to be a homeostatic mechanism that regulates effector cell function at the peak of the response.

While T cell exhaustion is usually associated with chronic infection, a recent study in the *L. major* mouse model suggests that the fate of exhausted T cells may be influenced by the events at the acute phase. Infection of otherwise resistant C57Bl/6 mice with arginase-deficient (*arg* ^−/−^) *L. major* causes chronic persistence of cutaneous lesions associated with exhaustion of specific CD4 T cells from the draining lymph nodes. The appearance of exhausted CD4 T cells at the chronic phase appears to be a consequence of the reduced primary CD4 response after infection with the transgenic/attenuated parasite [64]. Possibly, the curtailed acute response to *arg* ^−/−^ parasites precludes effective parasite elimination, which subsequently fosters the exhaustion of effector CD4 T cells due to antigen persistence. Alternatively, it cannot be ruled out that *arg*-deficient parasites are less fit in inducing APC activation and maturation. This would preclude efficient T cell activation and favour anergy. Given that the transcriptional profiles of anergic and exhausted T cells partially overlap [65], it is plausible that a naïve T cell primed under suboptimal conditions is more prone to undergo functional exhaustion at later stages of infection, unveiling a possible relationship between the two states of T cell responsiveness.

Immunoregulatory cytokines may also contribute to down-regulate T cell function by inducing or maintaining the states of exhaustion and anergy in protozoan infections [11], [66]. IL-10 impairs monocyte and dendritic cell maturation, inducing their deaths [67], which in turn may affect T cell effector function [68]. In the context of parasite infections, IL-10 can play a protective role by limiting tissue damage, as suggested by the increased mortality observed in *T. gondii* and *P. chabaudi* infections after IL-10 signalling neutralization [69], [70], or promote chronicity, as its neutralization during experimental visceral leishmaniasis improved CD4 T cell responses and led to disease resolution [71].

Finally, it is important to envision T cell exhaustion from a host–parasite co-evolution perspective. In this sense, T cell exhaustion may have evolved as a mechanism to avoid the immunopathology that would otherwise result from chronic pathogen persistence. Importantly, despite losing partial effector function, exhausted T cells may still apply some degree of immune pressure on the pathogen to attain the dynamic equilibrium that characterizes chronic infection [11], [72].

### T Cell Apoptosis As a Pathological Component of Protozoan Infections

**Figure 1:**
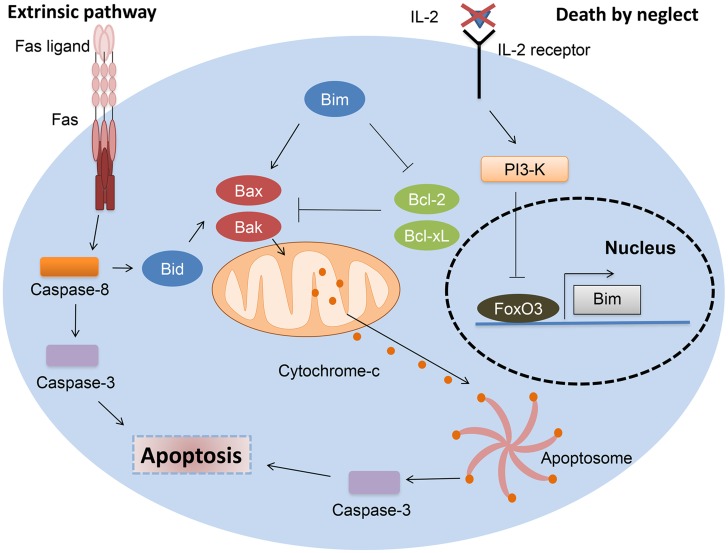
Mechanisms of T cell contraction after immune response resolution. T cell contraction after resolution of an immune response is usually accomplished through a combination of mitochondria- and death receptor–dependent mechanisms. As a result of T cell expansion, survival factors as IL-2 become scarce, and signalling through survival pathways, like the phosphoinositide 3-kinase (PI3-K)/Akt pathway, ceases, allowing FoxO3-dependent Bim induction. Bim promotes mitochondrial outer membrane permeabilization (MOMP) by relieving the inhibitory effect that antiapoptotic Bcl-2 and Bcl-xL exert on proapoptotic Bax and Bak. MOMP results in cytochrome-c release from the mitochondria, enabling activation of a supramolecular complex, the apoptosome that activates caspase-3. By processing numerous cellular substrates, activated caspase-3 ensures completion of the execution phase of apoptosis. T cell activation also induces Fas ligand expression in T cells, which, by engaging the death receptor Fas, enables caspase-8 activation at the death-inducing signalling complex (DISC). Caspase-8 then activates caspase-3. If the levels of caspase-8–activated caspase-3 are not sufficient to undertake apoptotic cell death, a mitochondrial amplification loop may occur through caspase-8–mediated Bid cleavage. This generates tBid, a proapoptotic Bcl-2 family member that promotes MOMP by activating Bax and Bak.

Apoptosis of T lymphocytes during the contraction phase of an immune response occurs through re-stimulation of activated T-cells in a process termed activation-induced cell death (AICD), or results from the absence of survival factors, known as activated T cell autonomous death (ACAD) or death by neglect [12]. AICD is usually accomplished through a death receptor-dependent mechanism. Upon activation, expression of death ligands, such as Fas ligand (FasL, CD95L) or TNF increases in T cells, allowing caspase-8 processing in the death-inducing signalling complex (DISC). In contrast, ACAD is mediated by the relative balance of the Bcl-2 family members [12]. In particular, the increased expression of the pro-apoptotic member Bim in T cells after cytokine deprival relieves the inhibitory effect anti-apoptotic Bcl-2 and Bcl-xL exert on Bax and Bak. This results in mitochondrial outer membrane permeabilization (MOMP) and apoptosome formation [12] (Figure 1).

Several studies demonstrate that T cell apoptosis occurs during natural or experimental infections with *Leishmania* spp. [73], *Toxoplasma gondii* [74], *Plasmodium* spp. [75], and *Trypanosoma cruzi* [76]. Furthermore, some studies showed evidence of augmented T cell responses and increased mouse survival after caspase inhibition during protozoan infection [77]–[79]. However, it is frequently hard to discern from published work whether T cell death is pathological or physiological [13], [80]. This is particularly notorious through the acute phase of infection, during which a high turnover of T cells is expected and reflects the emergence and shutdown of the primary response. Yet, even during the early stages, regulation of apoptosis may impact the mechanisms of T cell memory formation and compromise immunity during chronic infection [81]. In this section, we examined the findings that support a pathological component for T cell apoptosis during protozoan infections, proceeding either through a death receptor or mitochondria-dependent mechanism.

#### Involvement of death receptors

The first hint that death receptor–mediated apoptosis of T cells exerts a negative impact in the immune response during parasite disease came from studies revealing augmented T cell responses in *Fas*, *Faslg* (FasL), or *TNF-*deficient mice after parasite infection [82]–[86]. These studies, however, also evidenced the importance of death receptor signalling in the clearance of inflammatory infiltrates. Supporting these results is the finding that serum levels of FasL are elevated in patients chronically infected with *P. falciparum* [87], *T. cruzi* [88], and *L. donovani* [89].

Further analysis defined the kinetics of Fas and FasL expression and T lymphocyte apoptotic death during infection. Splenic CD4 and CD8 T cells start to express CD95 around the second to third week after murine *T. cruzi* infection, which correlates with their death by AICD [76], [90]. Treatment with an anti-FasL, but not anti-TNF or anti-TRAIL antibodies, could rescue both subsets from apoptosis, improving T cell effector functions and protecting mice from death [76], [90].

Importantly, mice vaccinated with an adenoviral vector expressing two *T. cruzi*–dominant epitopes presented improved CD8 T cell functionality and decreased parasitemia after parasite challenge, a phenotype attributed to the lack of CD95 expression in parasite-specific CD8 T cells [76]. Recently, the RIG-I–like receptor LGP2 was shown to repress CD95 expression in activated CD8 T cells in a murine model of West Nile virus infection [91]. While a potential role for LGP2 in parasitic infections remains to be addressed, this data has implications for vaccine design and how it could fine-tune the immune response with the aim to hamper death receptor signalling and improve T cell survival. Also, the decreased levels of parasitemia in immunized and infected animals may explain the lack of CD95 expression in CD8 T cells, due to lower immune activation [76].

Nevertheless, previous work has shown accelerated mice mortality after *T. cruzi* infection in the absence of Fas signalling [92], possibly due to excessive renal inflammation [93] and altered cytokine patterns that favour the expansion of a non-protective Th2 response [90]. Additionally, a recent study has revealed that a polymorphism in the *Fas* promoter is associated with protection in childhood malaria [94]. The protective *Fas* allele was associated with higher expression of CD95 in PBMCs, which was interpreted as facilitating T lymphocyte death and decreased immunopathology. These examples further demonstrate the dual roles played by death receptors, in particular Fas, during infection. On the one hand, death receptor triggering may compromise T cell immunity, but on the other hand, it prevents the pathogenic accumulation of activated T cell clones and limits tissue pathology.

Finally, some studies aimed to address the magnitude and functional properties of the T cell response after parasite infection of *Casp8* (caspase-8)-deficient mice or in the presence of caspase-8 inhibitors [77], [95]. These, however, yielded conflicting results and should be interpreted in view of the known role of caspase-8 in the activation of NF-κB after TCR triggering [96].

#### Death by neglect

The limitation of survival factors upon clonal expansion of activated T cells induces Bim expression in T cells, triggering the mitochondrial pathway of apoptosis, in a process known as death by neglect. By partnering with Fas-dependent mechanisms, Bim-mediated apoptosis ensures clearance of most effector T cell clones, yet allows the survival of a minute number of self-sustaining memory T cells [97].

Perhaps the clearest example of how Bim-mediated T cell apoptosis negatively affects the immune response during parasite infection comes from the *L. major* mouse model. While long-term immunity to *L. major* infection in resistant strains is thought to be dependent on the persistence of a small number of parasites in sheltered niches regulated by IL-10–producing effector or regulatory T cells [98], [99], sterile cure could be achieved after *Bim* ablation [100]. These mice exhibited increased numbers of parasite-specific CD4 T cells that produced IFN-γ at the infection site and draining lymph node and were protected from reinfection, suggesting again that interfering with T cell death may boost vaccine efficiency.

Recent evidence suggests that heightened expression of inflammatory mediators during the acute stages of infections exacerbates the contraction phase of the immune response, compromising the establishment of T cell memory [81]. During acute blood-stage *Plasmodium* infection in mice, a significant proportion of parasite-specific T cells undergo apoptotic demise. These could be saved by blocking IFN-γ signalling, but not TNF or Fas [75]. In a recent study, a *Plasmodium-*encoded homologue of the macrophage migration inhibitory factor (PMIF) was shown to potentiate the inflammatory response during acute blood-stage infection in mice. As a result, the differentiation of splenic T cells is diverted towards formation of short-lived terminal effector cells that die in a Bim-dependent manner [101]. *PMIF* ablation or IL-12/IFN-γ neutralization instead promoted the differentiation of long-lived memory T cells and ameliorated protection after reinfection [101]. Thus, a large proportion of T cell deaths during protozoan infection might be the result of differentiation of terminal effector T cells. In this sense, parasites exploit a host homeostatic pathway to curtail the magnitude and duration of the T cell response.

**Figure 2:**
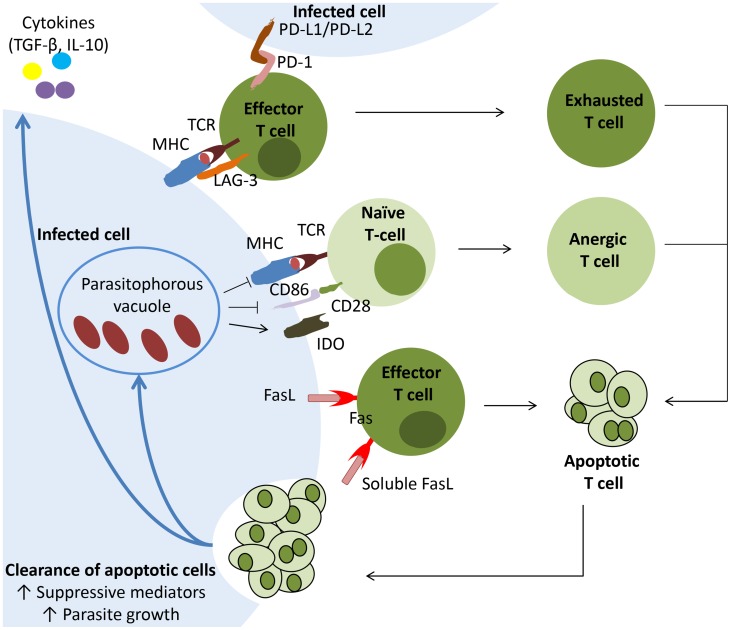
The uptake of apoptotic T lymphocytes by parasite-hosting phagocytes contributes to the remodelling of the parasite-hosting tissue as a bona fide protective niche. Increased rates of T cell apoptosis occur during parasite infection, mediated either by death receptor– or mitochondria-dependent mechanisms. Upon clearance, these apoptotic cells induce an alternative state of activation in phagocytes associated with production of suppressive mediators as TGF-β and IL-10, as well as promoting parasite growth. Suppressive cytokines act on effector T cells and, together with antigen persistence and inhibitory T cell receptors, induce exhaustion of these cells. Additionally, inhibition of antigen presentation and costimulation, acting along with suppressive cytokines or enzymes (as IDO, which catabolizes tryptophan), may render naïve T cells anergic and unresponsive throughout infection. Eventually effector, anergic, or exhausted T cells undergo programmed cell death, fuelling the pool of apoptotic corpses and aiding perpetuation of the suppressive state.

Finally, it is important to recognize that this increased rate of apoptotic T cell death is not without immunological consequences. Phagocyte internalization of dying cells suppresses production of inflammatory mediators but, instead, promotes expression of TGF-β and IL-10 [102]. Due to their suppressive effects on APCs functions, apoptotic cells may contribute to induce or maintain anergy and exhaustion in T cells, helping to perpetuate a state of down-regulated T cell function (Figure 2). Furthermore, internalization of apoptotic cells may actually fuel parasite growth inside macrophages, as observed for *T. cruzi* and *L. major* [103], [104].

### Concluding Remarks and Therapeutic Implications

The role of T cell immunity in controlling protozoan infection is clearly established, as demonstrated by parasite reactivation in the context of T cell immunodeficiency, such as co-infection with HIV. Intracellular parasites engage in complex and long-lasting interactions with the mammalian host during the full length of their developmental programs. Such intricate co-existence provides several targets for therapeutics to intersect the infection and ameliorate T cell function (Table 2).

**Table 2:** Impact of targeted inhibition of suppressive or apoptotic T cell pathways in the outcome of parasitic infection.

Suppressive pathway targeted	Parasite	Therapeutic approach	Infection outcome	Reference
T cell anergy	*Leishmania donovani*	Administration of an anti-LTβR mAb (blocks LIGHT binding to LTβR), started at the day of infection	Increased TNF production by hepatic CD4 T cells and promoted granuloma maturation and parasite clearance in the liver	[23]
	*Leishmania major*	Administration of an IDO inhibitor, initiated 14 days after infection	Increased CD4 T cell proliferation and reduced footpad swelling and parasite burden	[27]
	*Trypanosoma cruzi*	Treatment with inactive *T. cruzi* trans-sialidase, started at the day of infection	Treatment reverted the glycosylation status of CD8 T cells, decreased acute phase parasitemia and promoted mice survival	[39]
T cell exhaustion	*Toxoplasma gondii*	Anti-PD-L1 therapy, starting at 5 weeks post-infection	Augmented IFN-γ and granzyme B production by CD8 T cells and controlled *Toxoplasma* recrudescence	[48]
	*Leishmania donovani*	Anti-PD-L1 therapy, initiated at day 15 after infection	Rescued *L. donovani*–specific CD8 T cells from exhaustion with increased IFN-γ production and reduced splenic parasite burden	[24]
	*Plasmodium yoelii*	Anti-PD-L1 and anti-Lag3 therapy, starting at day 14 post-infection	Reinvigorated splenic CD4 and CD8 T cells, improved anti-*Plasmodium* humoral responses and decreased parasitemia	[60]
T cell apoptosis	*Trypanosoma cruzi*	Administration of zVAD (pan-caspase inhibitor), initiated at day 7 after infection	Reduced T cell apoptosis, promoted type 1 responses and reduced parasitemia	[77]
		Treatment with zLEHD (caspase-9 inhibitor), starting at 4 days post-infection	Protected mesenteric lymph node T cells from apoptosis and promoted their cytokine production	[78]
		Administration of an anti-FasL, starting at 11 days after infection	Therapy protected T cells from AICD, improved cytokine secretion and decreased parasitemia	[90]
		Therapy with zIETD (caspase-8 inhibitor), initiated 4 days after infection	Treatment inhibited T cell expansion and resulted in increased parasitemia	[95]
	*Plasmodium berghei*	Anti-IFN-γ treatment, daily, starting the first day after infection	Treatment prevented the deletion of parasite-specific CD4 T cells during acute phase of blood stage infection	[75]
		Anti-IFN-γ and anti-IL-12 treatment initiated 1 day before infection	Treatment promoted differentiation of long-lived memory CD4 T cells and decreased parasitemia	[75]

Nevertheless, some caveats should be highlighted as we set the stage to design future experiments in this area. First, experimental routes of infection do not always reflect accurately the events of a natural infection, particularly those early events associated with parasite establishment. Moreover, in humans, protozoan infection is often silent or less aggressive than mouse models, while also having a longer timeframe. An additional caveat in studies with human patients is the lack of analysis in deep tissues during the chronic phase, as often only peripheral blood is available. All this demands the introduction of alternative models that mimic the natural routes and more closely resemble human infection. An illustrative example comes from rats infected with *T. gondii* that develop a long-lasting chronic infection similar to human infection [105], [106]. However, this model remains poorly documented in the literature. Also, non-human primates (NHP) have proven to be faithful models of several human infectious diseases, particularly in AIDS research [107], [108]. Unfortunately, the use of NHP models in parasitic infections has been limited to pre-clinical drug or vaccine evaluation or clinical description of the infection [109]–[111], though recent studies have started to employ these models for more in-depth immunological descriptions [112], [113], (VR, ACS, ML, AO, RS, and JE, submitted manuscript).

The mechanisms of impaired T cell function that we reviewed here should be considered as complementary in effecting the immune escape responsible for parasite persistence and disease. In this context, the design of novel immunotherapies, such as therapeutic vaccines, may gain advantage in incorporating strategies that converge to restore immune competence.

**Five Key Papers in the Field**

1. Vasconcelos JR, Bruna-Romero O, Araujo AF, Dominguez MR, Ersching J, et al. (2012) Pathogen-induced proapoptotic phenotype and high CD95 (Fas) expression accompany a suboptimal CD8+ T-cell response: reversal by adenoviral vaccine. PLoS Pathog 8: e1002699. doi:10.1371/journal.ppat.1002699
2. Joshi T, Rodriguez S, Perovic V, Cockburn IA, Stager S (2009) B7-H1 blockade increases survival of dysfunctional CD8(+) T cells and confers protection against Leishmania donovani infections. PLoS Pathog 5: e1000431. doi:10.1371/journal.ppat.1000431
3. Reckling S, Divanovic S, Karp CL, Wojciechowski S, Belkaid Y, et al. (2008) Proapoptotic Bcl-2 family member Bim promotes persistent infection and limits protective immunity. Infect Immun 76: 1179–1185.
4. Bhadra R, Gigley JP, Weiss LM, Khan IA (2011) Control of Toxoplasma reactivation by rescue of dysfunctional CD8+ T-cell response via PD-1-PDL-1 blockade. Proc Natl Acad Sci U S A 108: 9196–9201.
5. Butler NS, Moebius J, Pewe LL, Traore B, Doumbo OK, et al. (2012) Therapeutic blockade of PD-L1 and LAG-3 rapidly clears established blood-stage Plasmodium infection. Nat Immunol 13: 188–195.

**Key Learning Points**

- The initial encounter between an intracellular protozoan parasite and the host's dedicated antigen-presenting cells is a multilayered interaction that often results in an inability of the latter to efficiently prime naïve antigen-specific T cells. This leads to delayed or anergic T cell responses, providing a window of time for parasite replication, dissemination, and sheltered establishment in the host.
- Chronic parasite persistence, acting along with immunosuppressive factors, profoundly affects the effector function of specific T cells, leading to their progressive loss of cytotoxic or helper activities in a phenomenon known as T cell exhaustion.
- By diverting T cell differentiation towards the formation of terminal effectors, intracellular parasites exacerbate the contraction phase of the T cell response, negatively influencing the establishment of a durable T cell memory and reducing the number of specific T cells available in the long-term.
- A delicate balance between T cell expansion and T cell death has to be attained in order to impose sufficient immune pressure on the parasite while also avoiding the immunopathology resulting from the accumulation of activated T cell clones.

## References

[1] Bhadra R, Gigley JP, Khan IA (2011) Cutting edge: CD40-CD40 ligand pathway plays a critical CD8-intrinsic and -extrinsic role during rescue of exhausted CD8 T cells. J Immunol 187: 4421–4425. 21949017 10.4049/jimmunol.1102319 PMC3197960

[2] Darrah PA, Patel DT, De Luca PM, Lindsay RW, Davey DF, et al (2007) Multifunctional TH1 cells define a correlate of vaccine-mediated protection against Leishmania major. Nat Med 13: 843–850. 17558415 10.1038/nm1592

[3] Schussek S, Trieu A, Apte SH, Sidney J, Sette A, et al (2013) Immunisation with AMA-1 confers sterile infection-blocking immunity against Plasmodium sporozoite challenge in a rodent model. Infect Immun 81: 3586–3599. 23836827 10.1128/IAI.00544-13 PMC3811765

[4] Yazdanbakhsh M, Sacks DL (2010) Why does immunity to parasites take so long to develop? Nat Rev Immunol 10: 80–81. 20183893 10.1038/nri2673 PMC3437742

[5] Sacks D, Sher A (2002) Evasion of innate immunity by parasitic protozoa. Nat Immunol 3: 1041–1047. 12407413 10.1038/ni1102-1041

[6] Rodrigues V, Cordeiro-da-Silva A, Laforge M, Ouaissi A, Silvestre R, et al (2012) Modulation of mammalian apoptotic pathways by intracellular protozoan parasites. Cell Microbiol 14: 325–333. 22168464 10.1111/j.1462-5822.2011.01737.x

[7] Fathman CG, Lineberry NB (2007) Molecular mechanisms of CD4+ T-cell anergy. Nat Rev Immunol 7: 599–609. 17612584 10.1038/nri2131

[8] Spence PJ, Langhorne J (2012) T cell control of malaria pathogenesis. Curr Opin Immunol 24: 444–448. 22658628 10.1016/j.coi.2012.05.003

[9] DosReis GA (2011) Evasion of immune responses by Trypanosoma cruzi, the etiological agent of Chagas disease. Braz J Med Biol Res 44: 84–90. 21243314 10.1590/s0100-879x2011007500005

[10] Engwerda CR, Ato M, Kaye PM (2004) Macrophages, pathology and parasite persistence in experimental visceral leishmaniasis. Trends Parasitol 20: 524–530. 15471704 10.1016/j.pt.2004.08.009

[11] Wherry EJ (2011) T cell exhaustion. Nat Immunol 12: 492–499. 21739672 10.1038/ni.2035

[12] Krammer PH, Arnold R, Lavrik IN (2007) Life and death in peripheral T cells. Nat Rev Immunol 7: 532–542. 17589543 10.1038/nri2115

[13] Gavrilescu LC, Denkers EY (2003) Apoptosis and the balance of homeostatic and pathologic responses to protozoan infection. Infect Immun 71: 6109–6115. 14573625 10.1128/IAI.71.11.6109-6115.2003 PMC219574

[14] Jenkins MK, Pardoll DM, Mizuguchi J, Chused TM, Schwartz RH (1987) Molecular events in the induction of a nonresponsive state in interleukin 2-producing helper T-lymphocyte clones. Proc Natl Acad Sci U S A 84: 5409–5413. 2955418 10.1073/pnas.84.15.5409 PMC298867

[15] Mueller DL (2010) Mechanisms maintaining peripheral tolerance. Nat Immunol 11: 21–27. 20016506 10.1038/ni.1817

[16] Chappert P, Schwartz RH (2010) Induction of T cell anergy: integration of environmental cues and infectious tolerance. Curr Opin Immunol 22: 552–559. 20869863 10.1016/j.coi.2010.08.005 PMC2981408

[17] Voisin MB, Buzoni-Gatel D, Bout D, Velge-Roussel F (2004) Both expansion of regulatory GR1+ CD11b+ myeloid cells and anergy of T lymphocytes participate in hyporesponsiveness of the lung-associated immune system during acute toxoplasmosis. Infect Immun 72: 5487–5492. 15322051 10.1128/IAI.72.9.5487-5492.2004 PMC517443

[18] Luft BJ, Kansas G, Engleman EG, Remington JS (1984) Functional and quantitative alterations in T lymphocyte subpopulations in acute toxoplasmosis. J Infect Dis 150: 761–767. 6238107 10.1093/infdis/150.5.761

[19] Haque S, Dumon H, Haque A, Kasper LH (1998) Alteration of intracellular calcium flux and impairment of nuclear factor-AT translocation in T cells during acute Toxoplasma gondii infection in mice. J Immunol 161: 6812–6818. 9862712

[20] McKee AS, Dzierszinski F, Boes M, Roos DS, Pearce EJ (2004) Functional inactivation of immature dendritic cells by the intracellular parasite Toxoplasma gondii. J Immunol 173: 2632–2640. 15294980 10.4049/jimmunol.173.4.2632

[21] Butcher BA, Kim L, Panopoulos AD, Watowich SS, Murray PJ, et al (2005) IL-10-independent STAT3 activation by Toxoplasma gondii mediates suppression of IL-12 and TNF-alpha in host macrophages. J Immunol 174: 3148–3152. 15749841 10.4049/jimmunol.174.6.3148

[22] Wei S, Marches F, Borvak J, Zou W, Channon J, et al (2002) Toxoplasma gondii-infected human myeloid dendritic cells induce T-lymphocyte dysfunction and contact-dependent apoptosis. Infect Immun 70: 1750–1760. 11895936 10.1128/IAI.70.4.1750-1760.2002 PMC127822

[23] Stanley AC, de Labastida Rivera F, Haque A, Sheel M, Zhou Y, et al (2011) Critical roles for LIGHT and its receptors in generating T cell-mediated immunity during Leishmania donovani infection. PLoS Pathog 7: e1002279 doi:10.1371/journal.ppat.1002279 21998581 10.1371/journal.ppat.1002279 PMC3188526

[24] Joshi T, Rodriguez S, Perovic V, Cockburn IA, Stager S (2009) B7-H1 blockade increases survival of dysfunctional CD8(+) T cells and confers protection against Leishmania donovani infections. PLoS Pathog 5: e1000431 doi:10.1371/journal.ppat.1000431 19436710 10.1371/journal.ppat.1000431 PMC2674929

[25] Martin DL, Weatherly DB, Laucella SA, Cabinian MA, Crim MT, et al (2006) CD8+ T-Cell responses to Trypanosoma cruzi are highly focused on strain-variant trans-sialidase epitopes. PLoS Pathog 2: e77 doi:10.1371/journal.ppat.0020077 16879036 10.1371/journal.ppat.0020077 PMC1526708

[26] Figueiredo AB, Serafim TD, Marques-da-Silva EA, Meyer-Fernandes JR, Afonso LC (2012) Leishmania amazonensis impairs DC function by inhibiting CD40 expression via A2B adenosine receptor activation. Eur J Immunol 42: 1203–1215. 22311598 10.1002/eji.201141926

[27] Makala LH, Baban B, Lemos H, El-Awady AR, Chandler PR, et al (2011) Leishmania major attenuates host immunity by stimulating local indoleamine 2,3-dioxygenase expression. J Infect Dis 203: 715–725. 21282196 10.1093/infdis/jiq095 PMC3072725

[28] Cortez M, Huynh C, Fernandes MC, Kennedy KA, Aderem A, et al (2011) Leishmania promotes its own virulence by inducing expression of the host immune inhibitory ligand CD200. Cell Host Microbe 9: 463–471. 21669395 10.1016/j.chom.2011.04.014 PMC3118640

[29] Gangneux JP, Poinsignon Y, Donaghy L, Amiot L, Tarte K, et al (2013) Indoleamine 2,3-dioxygenase activity as a potential biomarker of immune suppression during visceral leishmaniasis. Innate Immun 19: 564–568. 23413147 10.1177/1753425912473170

[30] Liehl P, Mota MM (2012) Innate recognition of malarial parasites by mammalian hosts. Int J Parasitol 42: 557–566. 22543040 10.1016/j.ijpara.2012.04.006

[31] Amino R, Thiberge S, Martin B, Celli S, Shorte S, et al (2006) Quantitative imaging of Plasmodium transmission from mosquito to mammal. Nat Med 12: 220–224. 16429144 10.1038/nm1350

[32] Chakravarty S, Cockburn IA, Kuk S, Overstreet MG, Sacci JB, et al (2007) CD8+ T lymphocytes protective against malaria liver stages are primed in skin-draining lymph nodes. Nat Med 13: 1035–1041. 17704784 10.1038/nm1628

[33] Belnoue E, Costa FT, Frankenberg T, Vigario AM, Voza T, et al (2004) Protective T cell immunity against malaria liver stage after vaccination with live sporozoites under chloroquine treatment. J Immunol 172: 2487–2495. 14764721 10.4049/jimmunol.172.4.2487

[34] Nganou-Makamdop K, van Gemert GJ, Arens T, Hermsen CC, Sauerwein RW (2012) Long term protection after immunization with P. berghei sporozoites correlates with sustained IFNgamma responses of hepatic CD8+ memory T cells. PLoS One 7: e36508 doi:10.1371/journal.pone.0036508 22563506 10.1371/journal.pone.0036508 PMC3341355

[35] Ing R, Segura M, Thawani N, Tam M, Stevenson MM (2006) Interaction of mouse dendritic cells and malaria-infected erythrocytes: uptake, maturation, and antigen presentation. J Immunol 176: 441–450. 16365437 10.4049/jimmunol.176.1.441

[36] Urban BC, Ferguson DJ, Pain A, Willcox N, Plebanski M, et al (1999) Plasmodium falciparum-infected erythrocytes modulate the maturation of dendritic cells. Nature 400: 73–77. 10403251 10.1038/21900

[37] Wykes MN, Liu XQ, Beattie L, Stanisic DI, Stacey KJ, et al (2007) Plasmodium strain determines dendritic cell function essential for survival from malaria. PLoS Pathog 3: e96 doi:10.1371/journal.ppat.0030096 17616976 10.1371/journal.ppat.0030096 PMC1904473

[38] Lundie RJ, Young LJ, Davey GM, Villadangos JA, Carbone FR, et al (2010) Blood-stage Plasmodium berghei infection leads to short-lived parasite-associated antigen presentation by dendritic cells. Eur J Immunol 40: 1674–1681. 20391433 10.1002/eji.200939265

[39] Freire-de-Lima L, Alisson-Silva F, Carvalho ST, Takiya CM, Rodrigues MM, et al (2010) Trypanosoma cruzi subverts host cell sialylation and may compromise antigen-specific CD8+ T cell responses. J Biol Chem 285: 13388–13396. 20106975 10.1074/jbc.M109.096305 PMC2859498

[40] Alcaide P, Fresno M (2004) The Trypanosoma cruzi membrane mucin AgC10 inhibits T cell activation and IL-2 transcription through L-selectin. Int Immunol 16: 1365–1375. 15314038 10.1093/intimm/dxh138

[41] Wing K, Onishi Y, Prieto-Martin P, Yamaguchi T, Miyara M, et al (2008) CTLA-4 control over Foxp3+ regulatory T cell function. Science 322: 271–275. 18845758 10.1126/science.1160062

[42] Fallarino F, Grohmann U (2011) Using an ancient tool for igniting and propagating immune tolerance: IDO as an inducer and amplifier of regulatory T cell functions. Curr Med Chem 18: 2215–2221. 21517758 10.2174/092986711795656027

[43] Haque A, Best SE, Amante FH, Mustafah S, Desbarrieres L, et al (2010) CD4+ natural regulatory T cells prevent experimental cerebral malaria via CTLA-4 when expanded in vivo. PLoS Pathog 6: e1001221 doi:10.1371/journal.ppat.1001221 21170302 10.1371/journal.ppat.1001221 PMC3000360

[44] Deaglio S, Dwyer KM, Gao W, Friedman D, Usheva A, et al (2007) Adenosine generation catalyzed by CD39 and CD73 expressed on regulatory T cells mediates immune suppression. J Exp Med 204: 1257–1265. 17502665 10.1084/jem.20062512 PMC2118603

[45] Jin HT, Jeong YH, Park HJ, Ha SJ (2011) Mechanism of T cell exhaustion in a chronic environment. BMB Rep 44: 217–231. 21524346 10.5483/BMBRep.2011.44.4.217

[46] Zajac AJ, Blattman JN, Murali-Krishna K, Sourdive DJ, Suresh M, et al (1998) Viral immune evasion due to persistence of activated T cells without effector function. J Exp Med 188: 2205–2213. 9858507 10.1084/jem.188.12.2205 PMC2212420

[47] Gigley JP, Bhadra R, Moretto MM, Khan IA (2012) T cell exhaustion in protozoan disease. Trends Parasitol 28: 377–384. 22832368 10.1016/j.pt.2012.07.001 PMC3768288

[48] Bhadra R, Gigley JP, Weiss LM, Khan IA (2011) Control of Toxoplasma reactivation by rescue of dysfunctional CD8+ T-cell response via PD-1-PDL-1 blockade. Proc Natl Acad Sci U S A 108: 9196–9201. 21576466 10.1073/pnas.1015298108 PMC3107287

[49] Bhadra R, Gigley JP, Khan IA (2012) PD-1-mediated attrition of polyfunctional memory CD8+ T cells in chronic toxoplasma infection. J Infect Dis 206: 125–134. 22539813 10.1093/infdis/jis304 PMC3415930

[50] Yi JS, Du M, Zajac AJ (2009) A vital role for interleukin-21 in the control of a chronic viral infection. Science 324: 1572–1576. 19443735 10.1126/science.1175194 PMC2736049

[51] Elsaesser H, Sauer K, Brooks DG (2009) IL-21 is required to control chronic viral infection. Science 324: 1569–1572. 19423777 10.1126/science.1174182 PMC2830017

[52] Hernandez-Ruiz J, Salaiza-Suazo N, Carrada G, Escoto S, Ruiz-Remigio A, et al (2010) CD8 cells of patients with diffuse cutaneous leishmaniasis display functional exhaustion: the latter is reversed, in vitro, by TLR2 agonists. PLoS Negl Trop Dis 4: e871 doi:10.1371/journal.pntd.0000871 21072232 10.1371/journal.pntd.0000871 PMC2970528

[53] Bustamante JM, Bixby LM, Tarleton RL (2008) Drug-induced cure drives conversion to a stable and protective CD8+ T central memory response in chronic Chagas disease. Nat Med 14: 542–550. 18425131 10.1038/nm1744 PMC3074975

[54] Albareda MC, Laucella SA, Alvarez MG, Armenti AH, Bertochi G, et al (2006) Trypanosoma cruzi modulates the profile of memory CD8+ T cells in chronic Chagas' disease patients. Int Immunol 18: 465–471. 16431876 10.1093/intimm/dxh387

[55] Laucella SA, Postan M, Martin D, Hubby Fralish B, Albareda MC, et al (2004) Frequency of interferon- gamma -producing T cells specific for Trypanosoma cruzi inversely correlates with disease severity in chronic human Chagas disease. J Infect Dis 189: 909–918. 14976609 10.1086/381682

[56] Albareda MC, Olivera GC, Laucella SA, Alvarez MG, Fernandez ER, et al (2009) Chronic human infection with Trypanosoma cruzi drives CD4+ T cells to immune senescence. J Immunol 183: 4103–4108. 19692645 10.4049/jimmunol.0900852 PMC3074976

[57] Chang DY, Song SH, You S, Lee J, Kim J, et al (2013) Programmed death-1 (PD-1)-dependent functional impairment of CD4 T cells in recurrent genital papilloma. Clin Exp Med E-pub ahead of print. doi:10.1007/s10238-013-0245-6 10.1007/s10238-013-0245-6 23824147

[58] Pallikkuth S, Fischl MA, Pahwa S (2013) Combination Antiretroviral Therapy With Raltegravir Leads to Rapid Immunologic Reconstitution in Treatment-Naive Patients With Chronic HIV Infection. J Infect Dis 208: 1613–1623. 23922374 10.1093/infdis/jit387 PMC3805240

[59] Day CL, Kaufmann DE, Kiepiela P, Brown JA, Moodley ES, et al (2006) PD-1 expression on HIV-specific T cells is associated with T-cell exhaustion and disease progression. Nature 443: 350–354. 16921384 10.1038/nature05115

[60] Butler NS, Moebius J, Pewe LL, Traore B, Doumbo OK, et al (2012) Therapeutic blockade of PD-L1 and LAG-3 rapidly clears established blood-stage Plasmodium infection. Nat Immunol 13: 188–195. 10.1038/ni.2180 PMC3262959 22157630

[61] Hafalla JC, Claser C, Couper KN, Grau GE, Renia L, et al (2012) The CTLA-4 and PD-1/PD-L1 inhibitory pathways independently regulate host resistance to Plasmodium-induced acute immune pathology. PLoS Pathog 8: e1002504 doi:10.1371/journal.ppat.1002504 22319445 10.1371/journal.ppat.1002504 PMC3271068

[62] Illingworth J, Butler NS, Roetynck S, Mwacharo J, Pierce SK, et al (2013) Chronic exposure to Plasmodium falciparum is associated with phenotypic evidence of B and T cell exhaustion. J Immunol 190: 1038–1047. 23264654 10.4049/jimmunol.1202438 PMC3549224

[63] Gutierrez FR, Mariano FS, Oliveira CJ, Pavanelli WR, Guedes PM, et al (2011) Regulation of Trypanosoma cruzi-induced myocarditis by programmed death cell receptor 1. Infect Immun 79: 1873–1881. 21357717 10.1128/IAI.01047-10 PMC3088162

[64] Mou Z, Muleme HM, Liu D, Jia P, Okwor IB, et al (2013) Parasite-Derived Arginase Influences Secondary Anti-Leishmania Immunity by Regulating Programmed Cell Death-1-Mediated CD4+ T Cell Exhaustion. J Immunol 190: 3380–3389. 23460745 10.4049/jimmunol.1202537 PMC3737427

[65] Wherry EJ, Ha SJ, Kaech SM, Haining WN, Sarkar S, et al (2007) Molecular signature of CD8+ T cell exhaustion during chronic viral infection. Immunity 27: 670–684. 17950003 10.1016/j.immuni.2007.09.006

[66] Jankovic D, Kugler DG, Sher A (2010) IL-10 production by CD4+ effector T cells: a mechanism for self-regulation. Mucosal Immunol 3: 239–246. 20200511 10.1038/mi.2010.8 PMC4105209

[67] Estaquier J, Ameisen JC (1997) A role for T-helper type-1 and type-2 cytokines in the regulation of human monocyte apoptosis. Blood 90: 1618–1625. 9269781

[68] Ouyang W, Rutz S, Crellin NK, Valdez PA, Hymowitz SG (2011) Regulation and functions of the IL-10 family of cytokines in inflammation and disease. Annu Rev Immunol 29: 71–109. 21166540 10.1146/annurev-immunol-031210-101312

[69] Freitas do Rosario AP, Lamb T, Spence P, Stephens R, Lang A, et al (2012) IL-27 promotes IL-10 production by effector Th1 CD4+ T cells: a critical mechanism for protection from severe immunopathology during malaria infection. J Immunol 188: 1178–1190. 22205023 10.4049/jimmunol.1102755 PMC3272378

[70] Jankovic D, Kullberg MC, Feng CG, Goldszmid RS, Collazo CM, et al (2007) Conventional T-bet(+)Foxp3(−) Th1 cells are the major source of host-protective regulatory IL-10 during intracellular protozoan infection. J Exp Med 204: 273–283. 17283209 10.1084/jem.20062175 PMC2118735

[71] Murray HW, Lu CM, Mauze S, Freeman S, Moreira AL, et al (2002) Interleukin-10 (IL-10) in experimental visceral leishmaniasis and IL-10 receptor blockade as immunotherapy. Infect Immun 70: 6284–6293. 12379707 10.1128/IAI.70.11.6284-6293.2002 PMC130311

[72] Virgin HW, Wherry EJ, Ahmed R (2009) Redefining chronic viral infection. Cell 138: 30–50. 19596234 10.1016/j.cell.2009.06.036

[73] Banerjee R, Kumar S, Sen A, Mookerjee A, Mukherjee P, et al (2011) TGF-beta-regulated tyrosine phosphatases induce lymphocyte apoptosis in Leishmania donovani-infected hamsters. Immunol Cell Biol 89: 466–474. 20856262 10.1038/icb.2010.108

[74] Gavrilescu LC, Denkers EY (2001) IFN-gamma overproduction and high level apoptosis are associated with high but not low virulence Toxoplasma gondii infection. J Immunol 167: 902–909. 11441097 10.4049/jimmunol.167.2.902

[75] Xu H, Wipasa J, Yan H, Zeng M, Makobongo MO, et al (2002) The mechanism and significance of deletion of parasite-specific CD4(+) T cells in malaria infection. J Exp Med 195: 881–892. 11927632 10.1084/jem.20011174 PMC2193727

[76] Vasconcelos JR, Bruna-Romero O, Araujo AF, Dominguez MR, Ersching J, et al (2012) Pathogen-induced proapoptotic phenotype and high CD95 (Fas) expression accompany a suboptimal CD8+ T-cell response: reversal by adenoviral vaccine. PLoS Pathog 8: e1002699 doi:10.1371/journal.ppat.1002699 22615561 10.1371/journal.ppat.1002699 PMC3355083

[77] Silva EM, Guillermo LV, Ribeiro-Gomes FL, De Meis J, Nunes MP, et al (2007) Caspase inhibition reduces lymphocyte apoptosis and improves host immune responses to Trypanosoma cruzi infection. Eur J Immunol 37: 738–746. 17295391 10.1002/eji.200636790

[78] de Meis J, Ferreira LM, Guillermo LV, Silva EM, Dosreis GA, et al (2008) Apoptosis differentially regulates mesenteric and subcutaneous lymph node immune responses to Trypanosoma cruzi. Eur J Immunol 38: 139–146. 18085669 10.1002/eji.200737582

[79] Begum-Haque S, Haque A, Kasper LH (2009) Apoptosis in Toxoplasma gondii activated T cells: the role of IFNgamma in enhanced alteration of Bcl-2 expression and mitochondrial membrane potential. Microb Pathog 47: 281–288. 19748565 10.1016/j.micpath.2009.09.004 PMC2771447

[80] Guillermo LV, Pereira WF, De Meis J, Ribeiro-Gomes FL, Silva EM, et al (2009) Targeting caspases in intracellular protozoan infections. Immunopharmacol Immunotoxicol 31: 159–173. 18785049 10.1080/08923970802332164

[81] Jameson SC, Masopust D (2009) Diversity in T cell memory: an embarrassment of riches. Immunity 31: 859–871. 20064446 10.1016/j.immuni.2009.11.007 PMC2957815

[82] Huang FP, Xu D, Esfandiari EO, Sands W, Wei XQ, et al (1998) Mice defective in Fas are highly susceptible to Leishmania major infection despite elevated IL-12 synthesis, strong Th1 responses, and enhanced nitric oxide production. J Immunol 160: 4143–4147. 9574511

[83] Lopes MF, Nunes MP, Henriques-Pons A, Giese N, Morse HC 3rd, et al (1999) Increased susceptibility of Fas ligand-deficient gld mice to Trypanosoma cruzi infection due to a Th2-biased host immune response. Eur J Immunol 29: 81–89. 9933089 10.1002/(SICI)1521-4141(199901)29:01<81::AID-IMMU81>3.0.CO;2-Y

[84] Conceicao-Silva F, Hahne M, Schroter M, Louis J, Tschopp J (1998) The resolution of lesions induced by Leishmania major in mice requires a functional Fas (APO-1, CD95) pathway of cytotoxicity. Eur J Immunol 28: 237–245. 9485203 10.1002/(SICI)1521-4141(199801)28:01<237::AID-IMMU237>3.0.CO;2-O

[85] Hu MS, Schwartzman JD, Yeaman GR, Collins J, Seguin R, et al (1999) Fas-FasL interaction involved in pathogenesis of ocular toxoplasmosis in mice. Infect Immun 67: 928–935. 9916110 10.1128/iai.67.2.928-935.1999 PMC96406

[86] Oliveira CF, Manzoni-de-Almeida D, Mello PS, Natale CC, Santiago Hda C, et al (2012) Characterization of chronic cutaneous lesions from TNF-receptor-1-deficient mice infected by Leishmania major. Clin Dev Immunol 2012: 865708. 22203861 10.1155/2012/865708 PMC3235446

[87] Kern P, Dietrich M, Hemmer C, Wellinghausen N (2000) Increased levels of soluble Fas ligand in serum in Plasmodium falciparum malaria. Infect Immun 68: 3061–3063. 10769016 10.1128/iai.68.5.3061-3063.2000 PMC97531

[88] Rodrigues V Jr, Agrelli GS, Leon SC, Silva Teixeira DN, Tostes S Jr, et al (2008) Fas/Fas-L expression, apoptosis and low proliferative response are associated with heart failure in patients with chronic Chagas' disease. Microbes Infect 10: 29–37. 18078776 10.1016/j.micinf.2007.09.015

[89] Eidsmo L, Wolday D, Berhe N, Sabri F, Satti I, et al (2002) Alteration of Fas and Fas ligand expression during human visceral leishmaniasis. Clin Exp Immunol 130: 307–313. 12390320 10.1046/j.1365-2249.2002.01976.x PMC1906528

[90] Guillermo LV, Silva EM, Ribeiro-Gomes FL, De Meis J, Pereira WF, et al (2007) The Fas death pathway controls coordinated expansions of type 1 CD8 and type 2 CD4 T cells in Trypanosoma cruzi infection. J Leukoc Biol 81: 942–951. 17261545 10.1189/jlb.1006643

[91] Suthar MS, Ramos HJ, Brassil MM, Netland J, Chappell CP, et al (2012) The RIG-I-like receptor LGP2 controls CD8(+) T cell survival and fitness. Immunity 37: 235–248. 22841161 10.1016/j.immuni.2012.07.004 PMC3910444

[92] Boyer MH, Hoff R, Kipnis TL, Murphy ED, Roths JB (1983) Trypanosoma cruzi: susceptibility in mice carrying mutant gene lpr (lymphoproliferation). Parasite Immunol 5: 135–142. 6405359 10.1111/j.1365-3024.1983.tb00731.x

[93] Oliveira GM, Masuda MO, Rocha NN, Schor N, Hooper CS, et al (2009) Absence of Fas-L aggravates renal injury in acute Trypanosoma cruzi infection. Mem Inst Oswaldo Cruz 104: 1063–1071. 20140366 10.1590/s0074-02762009000800002

[94] Schuldt K, Kretz CC, Timmann C, Sievertsen J, Ehmen C, et al (2011) A -436C>A polymorphism in the human FAS gene promoter associated with severe childhood malaria. PLoS Genet 7: e1002066 doi:10.1371/journal.pgen.1002066 21625619 10.1371/journal.pgen.1002066 PMC3098189

[95] Silva EM, Guillermo LV, Ribeiro-Gomes FL, De Meis J, Pereira RM, et al (2005) Caspase-8 activity prevents type 2 cytokine responses and is required for protective T cell-mediated immunity against Trypanosoma cruzi infection. J Immunol 174: 6314–6321. 15879131 10.4049/jimmunol.174.10.6314

[96] Su H, Bidere N, Zheng L, Cubre A, Sakai K, et al (2005) Requirement for caspase-8 in NF-kappaB activation by antigen receptor. Science 307: 1465–1468. 15746428 10.1126/science.1104765

[97] Hughes PD, Belz GT, Fortner KA, Budd RC, Strasser A, et al (2008) Apoptosis regulators Fas and Bim cooperate in shutdown of chronic immune responses and prevention of autoimmunity. Immunity 28: 197–205. 18275830 10.1016/j.immuni.2007.12.017 PMC2270348

[98] Pagan AJ, Peters NC, Debrabant A, Ribeiro-Gomes F, Pepper M, et al (2012) Tracking antigen-specific CD4(+) T cells throughout the course of chronic Leishmania major infection in resistant mice. Eur J Immunol 43: 427–438. 23109292 10.1002/eji.201242715 PMC4086308

[99] Belkaid Y, Piccirillo CA, Mendez S, Shevach EM, Sacks DL (2002) CD4+CD25+ regulatory T cells control Leishmania major persistence and immunity. Nature 420: 502–507. 12466842 10.1038/nature01152

[100] Reckling S, Divanovic S, Karp CL, Wojciechowski S, Belkaid Y, et al (2008) Proapoptotic Bcl-2 family member Bim promotes persistent infection and limits protective immunity. Infect Immun 76: 1179–1185. 18086806 10.1128/IAI.01093-06 PMC2258821

[101] Sun T, Holowka T, Song Y, Zierow S, Leng L, et al (2012) A Plasmodium-encoded cytokine suppresses T-cell immunity during malaria. Proc Natl Acad Sci U S A 109: E2117–2126. 22778413 10.1073/pnas.1206573109 PMC3411961

[102] Erwig LP, Henson PM (2007) Immunological consequences of apoptotic cell phagocytosis. Am J Pathol 171: 2–8. 17591947 10.2353/ajpath.2007.070135 PMC1941587

[103] Freire-de-Lima CG, Nascimento DO, Soares MB, Bozza PT, Castro-Faria-Neto HC, et al (2000) Uptake of apoptotic cells drives the growth of a pathogenic trypanosome in macrophages. Nature 403: 199–203. 10646605 10.1038/35003208

[104] Ribeiro-Gomes FL, Otero AC, Gomes NA, Moniz-De-Souza MC, Cysne-Finkelstein L, et al (2004) Macrophage interactions with neutrophils regulate Leishmania major infection. J Immunol 172: 4454–4462. 15034061 10.4049/jimmunol.172.7.4454

[105] Zenner L, Estaquier J, Darcy F, Maes P, Capron A, et al (1999) Protective immunity in the rat model of congenital toxoplasmosis and the potential of excreted-secreted antigens as vaccine components. Parasite Immunol 21: 261–272. 10320624 10.1046/j.1365-3024.1999.00229.x

[106] Zenner L, Foulet A, Caudrelier Y, Darcy F, Gosselin B, et al (1999) Infection with Toxoplasma gondii RH and Prugniaud strains in mice, rats and nude rats: kinetics of infection in blood and tissues related to pathology in acute and chronic infection. Pathol Res Pract 195: 475–485. 10448664 10.1016/S0344-0338(99)80051-X

[107] Hurtrel B, Petit F, Arnoult D, Muller-Trutwin M, Silvestri G, et al (2005) Apoptosis in SIV infection. Cell Death Differ 12 (Suppl 1) 979–990. 15818408 10.1038/sj.cdd.4401600

[108] Evans DT, Silvestri G (2013) Nonhuman primate models in AIDS research. Curr Opin HIV AIDS 8: 255–261. 23615116 10.1097/COH.0b013e328361cee8 PMC3987953

[109] Porrozzi R, Pereira MS, Teva A, Volpini AC, Pinto MA, et al (2006) Leishmania infantum-induced primary and challenge infections in rhesus monkeys (Macaca mulatta): a primate model for visceral leishmaniasis. Trans R Soc Trop Med Hyg 100: 926–937. 16455120 10.1016/j.trstmh.2005.11.005

[110] Grimaldi G Jr (2008) The utility of rhesus monkey (Macaca mulatta) and other non-human primate models for preclinical testing of Leishmania candidate vaccines. Mem Inst Oswaldo Cruz 103: 629–644. 19057811 10.1590/s0074-02762008000700002

[111] Moreno A, Cabrera-Mora M, Garcia A, Orkin J, Strobert E, et al (2013) Plasmodium coatneyi in rhesus macaques replicates the multisystemic dysfunction of severe malaria in humans. Infect Immun 81: 1889–1904. 23509137 10.1128/IAI.00027-13 PMC3676004

[112] Trott KA, Richardson A, Hudgens MA, Abel K (2013) Immune Activation and Regulation in Simian Immunodeficiency Virus-Plasmodium fragile-Coinfected Rhesus Macaques. J Virol 87: 9523–9537. 23785209 10.1128/JVI.00861-13 PMC3754117

[113] de-Campos SN, Souza-Lemos C, Teva A, Porrozzi R, Grimaldi G Jr (2010) Systemic and compartmentalised immune responses in a Leishmania braziliensis-macaque model of self-healing cutaneous leishmaniasis. Vet Immunol Immunopathol 137: 149–154. 20546932 10.1016/j.vetimm.2010.04.009

